# Congenital Myasthenic Syndrome With Adult Onset Due to the Novel Heterozygous c.1399_1404del Variant in the Downstream of Tyrosine Kinase-7 (DOK7): A Case Report

**DOI:** 10.7759/cureus.81690

**Published:** 2025-04-04

**Authors:** Josef Finsterer

**Affiliations:** 1 Neurology Department, Neurology and Neurophysiology Center, Vienna, AUT

**Keywords:** 3, 4-diaminopyridine, congenital myasthenic syndrome, dok-7, post-synaptic, salbutamol

## Abstract

Although mutations in the downstream of tyrosine kinase-7 (DOK7) are one of the most common causes of congenital myasthenic syndrome (CMS) in children and adults, CMS in adults due to the heterozygous variant c.1399_1404del in DOK7 has not yet been reported.

A 63-year-old woman had developed bilateral eyelid ptosis at the age of 50, followed by dysphagia shortly thereafter. At the age of 60, in addition to dysphagia, she developed dysarthria and decreased cough. Her sister had a history of right eyelid ptosis. Myasthenia gravis and myasthenic syndrome, motor neuron disease, and myopathy were ruled out in the index patient. Pyridostigmine, steroids, azathioprine, methotrexate, mycophenolate mofetil, and immunoglobulins were either ineffective or complicated by side effects. Genetic testing at the age of 61 years revealed the variants c.1399_1404del and c.54+32_54+33del in DOK7. Late-onset CMS was diagnosed, and salbutamol and later 3,4-diaminopyridine (3,4-DAP) were started, both of which showed a positive effect.

This case shows that the DOK7 variant c.1399_1404del is probably pathogenic and responsible for late-onset CMS, either alone or together with the previously reported benign variant c.54+32_54+33del. Salbutamol in combination with 3,4-DAP could be beneficial in patients carrying the c.1399_1404del mutation in DOK7.

## Introduction

Congenital myasthenic syndromes (CMS) are a hereditary, clinically and genetically heterogeneous group of neuromuscular transmission disorders caused by mutations in genes encoding different proteins that form the architecture and function of the neuromuscular endplate [1-3]. Pathophysiologically, four different types of CMS are distinguished, depending on where the mutated protein is located within the endplate structure [1]. These include presynaptic CMS, synaptic CMS, postsynaptic CMS, and glycosylation defects [1-3]. The phenotype of the different CMS is heterogeneous, as not only the onset but also the clinical presentation, treatment, and outcome of the disease vary greatly [1-3]. The most common symptoms of CMS include weakness of the arm or leg muscles, double vision, drooping eyelids, and problems with speech, chewing, swallowing, and breathing [1,2]. Rarer manifestations include skeletal deformities, facial dysmorphisms, hypoacusis, seizures, or renal insufficiency.

One of the most frequently mutated genes in postsynaptic CMS is downstream of tyrosine kinase-7 (DOK7) [4]. DOK7 is responsible for the phosphorylation of the β-subunit of the acetylcholine receptor (AChR) and the maintenance of AChR site density [5]. At the neuromuscular endplate, DOK7 enhances the phosphorylation of muscle-specific kinase (MuSK) and induces the clustering of AChRs [6]. However, there are patients carrying DOK7 variants in which the endplates have normal AChR-β subunit phosphorylation, and AChR density on the remaining folds of the junction is normal [5]. DOK7 is essential not only for maintaining the size but also the structural integrity of the endplate [5]. The profound structural changes at the endplates likely contribute significantly to the reduced safety margin of neuromuscular transmission [5]. Some DOK7 mutations are complex and only identifiable in cloned complementary DNA [5]. The phenotype of DOK7-related CMS is highly variable [5]. Some patients with DOK7 mutations phenotypically mimic congenital muscular dystrophy [7]. The most effective treatment for DOK7-associated CMS is salbutamol [4]. However, ephedrine, 3,4-diaminopyridine (3,4-DAP) (amifampridine), and fluoxetine are also effective in individual patients [8]. In a woman with subacute onset of myasthenia during pregnancy due to the DOK7 mutation, fluoxetine was partially helpful [9]. CMS in adulthood caused by the heterozygous variant c.1399_1404del in DOK7 has not been reported [10].

## Case presentation

The index patient is a 63-year-old woman who developed bilateral ptosis at the age of 50, which was treated with laser therapy (Table 1), followed by dysphagia, initially for solid food only, later also for liquids. There was a history of arterial hypertension, hypercholesterolemia, type-II diabetes mellitus, bilateral cataract, moderate obstructive sleep apnea syndrome (OSAS), hiatal hernia, osteoporosis with vertebral fracture D12-L1, cervical spondylarthritis, multiple lumbar stenosis, hepatitis-C infection with eradication therapy, hepatitis-B, and left knee arthroplasty. Family history included ptosis of the right eyelid (sister) and Crohn's disease (sister).

**Table 1:** Timeline of the patient’s disease course DAP: diaminopyridine; AChR: acetylcholine receptor; EMG: electromyography; MuSK: muscle-specific kinase; DOK7: downstream of tyrosine kinase-7

Age (years/month)	Diagnosis/event	Treatment/consequence
50	Bilateral ptosis	Laser therapy
50	Dysphagia for solids and later liquids, hypertonic cardia	Dilatation (partial benefit)
60/3	Dysphagia, dysarthria, myopathic facial EMG, AChR, MuSK negative	Pyridostigmine recommended
60/11	Persistence of symptoms	Immunoglobulins (moderate benefit), prednisolone (initial benefit)
61/3	Side effects of steroids	Prednisolone
61/3	Clinical worsening, double vision	Prednisolone ­
61/4	Persistence of symptoms	Immunoglobulins
61/5	Persistence of symptoms	Azathioprine, prednisolone
61/5	Transaminases ­, dysarthria, occasional dysphagia, urine incontinence, cramps, tremor, repetitive stimulation normal, AChR, MuSK negative, lactate ­, no PABN1 mutation, DOK7 mutation detected	Azathioprine withdrawn, prednisolone ­
61/11	Symptoms worsen after SARS-CoV-2 infection	Prednisolone
61/12	The state before the SARS-CoV-2 infection is reached again	Mycophenolate mofetil
62/1	Transaminases increase­, myalgias	Mycophenolate withdrawn, pyridostigmine, methotrexate
62/4	Myalgias persist	Methotrexate withdrawn, salbutamol started
62/7	Dysarthria, dysphagia improve, expectoration impaired	3,4-DAP started, prednisolone ­
62/11	Further improvement of bulbar symptoms, cramps	Ozone for lower limb muscles
63/1	Mild ptosis, no bulbar symptoms	Continuation of 3,45-DAP, salbutamol

**Figure 1:**
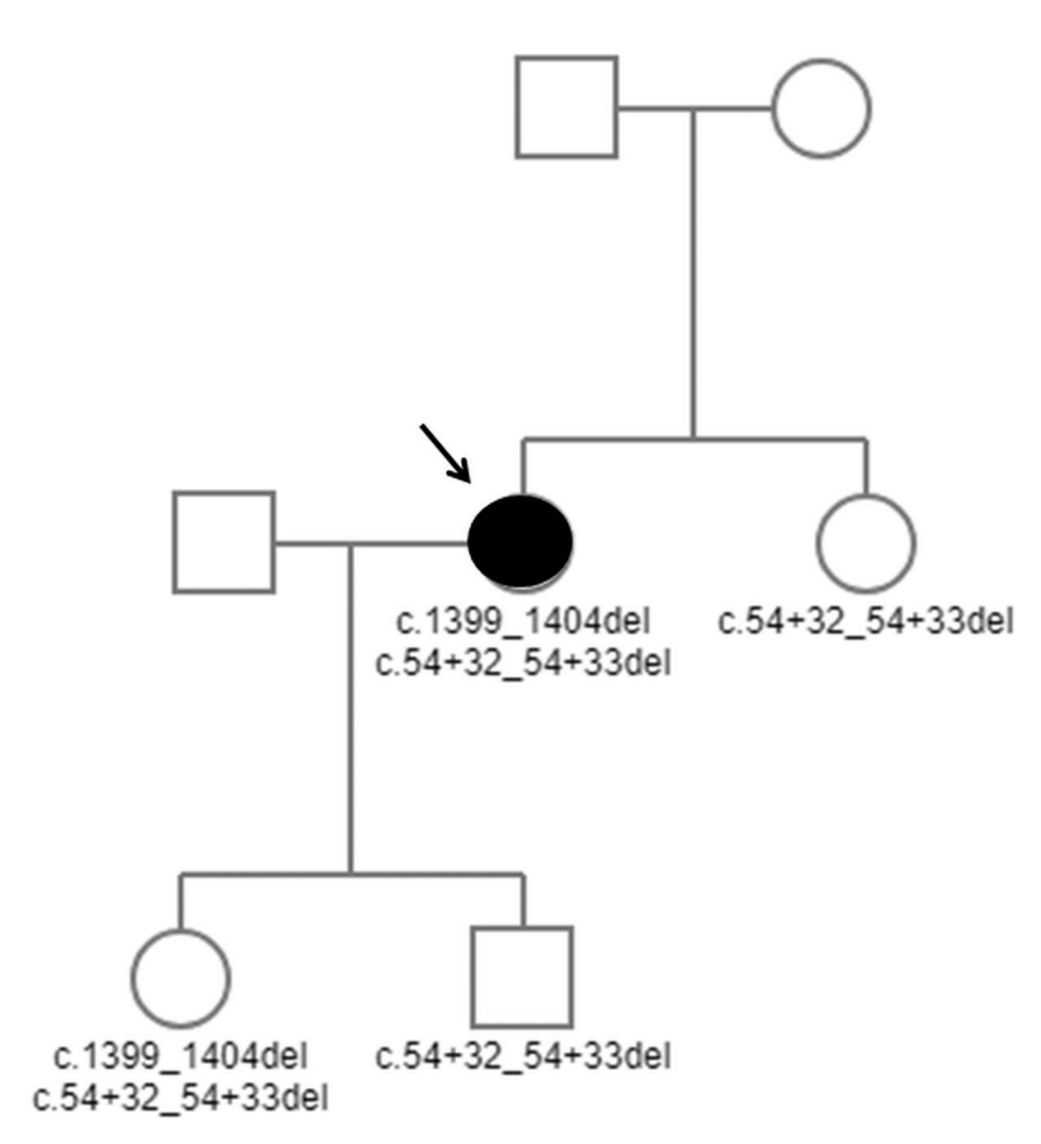
Index family tree showing the clinically affected index patient and her two children, both of whom were clinically unaffected but had different DOK7 mutation statuses (squares: men, circles: women, filled circle: clinically affected index patient, unfilled circles/squares: clinically unaffected) DOK7: downstream of tyrosine kinase-7

Initially, the dysphagia was attributed to hypertension of the esophageal-gastric junction with incomplete relaxation, with non-conductive peristaltic waves detected in 10% of the patients on manometry. For this reason, pneumatic dilatation was performed at the age of 55 but was only partially successful. Since then, phases of well-being alternated with episodes of gagging and dysphagia with solid food. At the age of 60, she developed dysarthria in addition to dysphagia, which is why myasthenia gravis was suspected. However, the antibodies against postsynaptic AChR and MuSK were negative. Thyroid function tests were normal, as was cerebral magnetic resonance imaging (MRI), but electromyography (EMG) of the orbicularis oris muscle was myopathic. Despite negative evidence of myasthenia antibodies, pyridostigmine was prescribed. Five months later, she underwent treatment with intravenous immunoglobulins (IVIGs), which was only moderately successful, followed by prednisolone (50 mg/d), which only helped initially, but not in the long term. Computed tomography (CT) of the chest was negative for a thymoma. Due to severe side effects, prednisolone was reduced to 25 mg/day four months later. Asthenia and diplopia occurred under this regimen, so prednisolone was increased again to 37.5 mg/d. One month later, she received a second IVIG cycle, which was canceled due to vomiting. Genetic testing by means of Sanger sequencing of exons 1-7 of the DOK7 gene revealed the heterozygous mutation c.1399_1404del and c.54+32_54+33del in DOK7 (Figure 1). These variants were also found in her asymptomatic daughter. Her son and sister carried only the c.54+32_54+33del variant in DOK7 (Figure 1). The variant c.1399_1404del led to the amino acid change G468_P469del. Despite these findings, azathioprine was started but had to be discontinued one month later due to increasing transaminases (Table 1). Prednisolone was reduced to 25 mg/d.

Under this regimen, rhinolalia persisted, and moderate generalized asthenia, urinary incontinence, occasional dysphagia, muscle cramps, arthralgias, and tremor occurred. Antibodies to AChR (determined by means of a radioimmunoassay) and MuSK (determined by means of an immunofluorescence assay) remained negative, and the search for mutations in PABN1 was negative, ruling out oculopharyngeal muscular dystrophy. EMG of the orbicularis oris was again myopathic, and repetitive nerve stimulation (Desmedt test) was normal (decrement <10%). The serum lactate was elevated to 4.4 mmol/L. At the age of 61 years, prednisolone was reduced to 2.5 mg/day, which led to an improvement in veiling, but dysphagia and dysarthria persisted. One month later, she received mycophenolate mofetil, which had to be discontinued after one month due to increasing transaminases and myalgias. At the age of 62, pyridostigmine was tried again, but this was not tolerated, so methotrexate (MTX) 7.5 to 10 mg/d was started. Prednisolone was increased again to 5 mg, and lamivudine for hepatitis B virus (HBV) was added. At the age of 62 years and four months, MTX was reduced due to arthralgia, and salbutamol (6 mg/d) was started. Under salbutamol, there was an improvement in dysphagia and dysarthria, but the difficulty in coughing remained, the tremor increased, and involuntary movements occurred. At the age of 62 years and seven months, 3,4-DAP was added, which led to a further improvement. At the age of 63 years and one month, symptoms had further improved (Table 1), but the patient still wanted to reduce salbutamol and 3,4-DAP because of side effects such as tremor, tachycardia, muscle spasms (salbutamol), and weakness, fatigue, and blurred vision (3,4-DAP). Her current medication included prednisolone (7.5 mg/d), 3,4-DAP (10 mg/d), salbutamol (8 mg/d), lansoprazole, olmesartan/HCT, and teriparatide.

## Discussion

The patient is interesting in several respects. First, she carried a novel heterozygous mutation in DOK7. The variant c.1399_1404del in DOK7 has not been previously described, but the clinical presentation suggests that the variant is potentially pathogenic. Arguments for the pathogenicity of the variant are the clinical presentation with ptosis, dysphagia, dysarthria, and reduced cough, as well as the amino acid change caused by the mutation. Heterozygosity of the variant is not an argument against pathogenicity, as several patients with heterozygous DOK7 variants have been reported. Whether urinary incontinence, tremor, and muscle spasms were a consequence of the DOK7 variant remains speculative and requires further investigation. Tremor is a common side effect of salbutamol. Muscle cramps could also be attributed to salbutamol. Neurogenic bladder is a common manifestation of Lambert-Eaton myasthenia syndrome, which was excluded in the index patient. Whether the c.54+32_54+33del variant contributed to the pathogenicity of c.1399_1404del remains speculative, as the c.54+32_54+33del variant was previously classified as benign [11].

Second, salbutamol and 3,4-DAP were effective for dysphagia, dysarthria, and reduced cough. Salbutamol has repeatedly been shown to be beneficial for motor symptoms in DOK7-associated CMS patients [12]. There is also evidence from a mouse model that salbutamol increases the number of neuromuscular junctions [13]. In addition, there is a report of a patient with the homozygous variant c.1124_1127dup who responded favorably to fluoxetine [9]. In addition, there is a report of a pediatric patient with a DOK7 variant who developed rapidly progressive limb-girdle weakness requiring the use of a wheelchair for activities of daily living, which improved within four weeks of starting treatment with albuterol [14]. Albuterol has also been shown to be beneficial in four other patients with DOK7 mutations [14]. In a study of 12 patients with DOK7-associated CMS, a positive effect of ephedrine was observed [15]. A gradual improvement in quantitative myasthenia score, mobility score, and activities of daily living was documented in these patients [15]. The improvement in weakness mainly affected the muscles of the proximal limbs [15]. The administration of acetylcholinesterase inhibitors can worsen symptoms in DOK7-related CMS [16], which is why they should not be administered to DOK7 mutation carriers.

Third, the patient was initially misdiagnosed as having hypertension of the esophageal-gastric junction with incomplete relaxation and later as an acquired transmission disorder (myasthenia gravis) for 12 years and treated accordingly with prednisolone, IVIGs, azathioprine, mycophenolate mofetil, and MTX with no appreciable benefit. Misdiagnosis of CMS is common because the various phenotypic manifestations of CMS mimic motor neuron disease, myopathies, and acquired transmission disorders. In a French study of 235 CMS patients, the correct diagnosis was made in 139 patients in adulthood, of whom 110 patients had their first symptoms before the age of 18 years. A single patient with DOK7-associated CMS was misdiagnosed as having congenital myopathy for 25 years [17,18]. In a patient with late-onset ptosis, dysphagia, and dysarthria, but without AChR, MuSK, and LRP4 antibodies, without thymoma, and without decrement, but with myopathic EMG, CMS should be considered and excluded.

Fourth, the presence of diabetes, hyperlipidemia, cataract, hiatal hernia, myopathy, osteoporosis, and lactic acidosis suggests a second problem in addition to the DOK7 mutation. The combination of these phenotypic features particularly suggests a mitochondrial disorder, but this remains speculative and requires further biochemical and genetic studies. Only myopathy may be a feature of CMS clinically and in biopsy [19]. Other phenotypic features of CMS may include dysmorphism, hyperextensible joints, generalized hypotonia, positive Gower's sign, anserine ambulation, and the transient presence of antibodies to AChR antibodies [19].

Myopathic facial muscle EMG and elevated serum lactate levels in the index patient are consistent with previous reports [20]. In a study of 23 patients with early-onset CMS due to DOK7 variants, muscle biopsy showed areas without oxidative enzyme activity in six of 17 muscle biopsies [20]. However, as in the index patient, muscle biopsy more often showed non-specific findings than myopathic changes [20]. A diagnostic clue suggesting CMS and not myopathy in this study was the discrepancy between muscle imaging or histological findings compared to the degree of muscle weakness [20].

Limitations of the study are that the results of whole exome sequencing (WES) and sequencing of mitochondrial DNA (mtDNA) are not available, that functional and biochemical studies were not carried out to document the pathogenicity of the detected DOK7 variant, that the patient was treated with glucocorticoids despite knowledge of the DOK7 variant and the presence of diabetes, that LRP4 antibodies and titin antibodies were not determined, that muscle MRI was not performed, and that, with the exception of salbutamol and 3,4-DAP, no other agents commonly administered and of benefit in DOK7 carriers have not yet been tried. Another limitation is that the effect of the different therapies used was only assessed by the treating physicians without objectively measuring the changes.

## Conclusions

In summary, this case shows that the DOK7 variant c.1399_1404del is likely pathogenic based on clinical presentation and responsible for late-onset CMS, either alone or in combination with the previously reported harmless variant c.54+32_54+33del. The study also suggests that salbutamol alone or in combination with 3,4-DAP could be beneficial with regard to the symptoms of dysphagia, dysarthria, and reduced cough in carriers of this DOK7 variant. Clinicians should be aware that bulbar symptoms, along with ptosis and respiratory muscle weakness, may be due not only to motor neuron disease, myopathy, or acquired transmission disorders, but also to CMS, even in patients with late-onset symptoms. It is also necessary that long-term follow-up and larger studies are carried out.

## References

[1] Henehan L, Beeson D, Palace J (2024). Congenital myasthenic syndromes. Pract Neurol.

[2] Finsterer J (2019). Congenital myasthenic syndromes. Orphanet J Rare Dis.

[3] Engel AG, Shen XM, Selcen D, Sine SM (2015). Congenital myasthenic syndromes: pathogenesis, diagnosis, and treatment. Lancet Neurol.

[4] Ziaadini B, Ghaderi Yazdi B, Dirandeh E (2024). DOK7 congenital myasthenic syndrome: case series and review of literature. BMC Neurol.

[5] Selcen D, Milone M, Shen XM, Harper CM, Stans AA, Wieben ED, Engel AG (2008). Dok-7 myasthenia: phenotypic and molecular genetic studies in 16 patients. Ann Neurol.

[6] Zhang S, Ohkawara B, Ito M (2023). A mutation in DOK7 in congenital myasthenic syndrome forms aggresome in cultured cells, and reduces DOK7 expression and MuSK phosphorylation in patient-derived iPS cells. Hum Mol Genet.

[7] Mahjneh I, Lochmüller H, Muntoni F, Abicht A (2013). DOK7 limb-girdle myasthenic syndrome mimicking congenital muscular dystrophy. Neuromuscul Disord.

[8] Palace J (2012). DOK7 congenital myasthenic syndrome. Ann N Y Acad Sci.

[9] Santos M, Cruz S, Peres J (2018). DOK7 myasthenic syndrome with subacute adult onset during pregnancy and partial response to fluoxetine. Neuromuscul Disord.

[10] Hlobal variome shared LOVD. Dok 7 (docking protein-7 (2025). Global Variome shared LOVD DOK7 (docking protein 7). https://databases.lovd.nl/shared/variants/DOK7.

[11] (2025). National Center for Biotechnology Information. ClinVar; [VCV000701812.12]. https://www.ncbi.nlm.nih.gov/clinvar/variation/VCV000701812.12.

[12] Khadilkar S, Bhutada A, Nallamilli B, Hegde M (2015). Limb girdle weakness responding to salbutamol: an Indian family with DOK7 mutation. Indian Pediatr.

[13] Webster RG, Vanhaesebrouck AE, Maxwell SE (2020). Effect of salbutamol on neuromuscular junction function and structure in a mouse model of DOK7 congenital myasthenia. Hum Mol Genet.

[14] Tsao CY (2016). Effective treatment with albuterol in DOK7 congenital myasthenic syndrome in children. Pediatr Neurol.

[15] Lashley D, Palace J, Jayawant S, Robb S, Beeson D (2010). Ephedrine treatment in congenital myasthenic syndrome due to mutations in DOK7. Neurology.

[16] Lozowska D, Ringel SP, Winder TL, Liu J, Liewluck T (2015). Anticholinesterase therapy worsening head drop and limb weakness due to a novel DOK7 mutation. J Clin Neuromuscul Dis.

[17] Theuriet J, Masingue M, Behin A (2024). Congenital myasthenic syndromes in adults: clinical features, diagnosis and long-term prognosis. Brain.

[18] Johnson A, Subramony SH, Chuquilin M (2018). Delayed diagnosis of DOK7 congenital myasthenic syndrome: case report and literature review. Neurol Clin Pract.

[19] Vallepu SB, Dhamija K, Rajan GK, Panchal T, Saran RK, Roshan S (2025). Phenotypic variability in congenital myasthenic syndrome with GFPT1 mutation. Acta Neurol Belg.

[20] Klein A, Pitt MC, McHugh JC (2013). DOK7 congenital myasthenic syndrome in childhood: early diagnostic clues in 23 children. Neuromuscul Disord.

